# Anodal Transcranial Direct Current Stimulation Does Not Facilitate Dynamic Balance Task Learning in Healthy Old Adults

**DOI:** 10.3389/fnhum.2017.00016

**Published:** 2017-01-31

**Authors:** Elisabeth Kaminski, Maike Hoff, Viola Rjosk, Christopher J. Steele, Christopher Gundlach, Bernhard Sehm, Arno Villringer, Patrick Ragert

**Affiliations:** ^1^Department of Neurology, Max Planck Institute for Human Cognitive and Brain SciencesLeipzig, Germany; ^2^Department of Psychiatry, Cerebral Imaging Centre, Douglas Mental Health Institute, McGill UniversityMontreal, QC, Canada; ^3^Faculty of Psychology, Department of Experimental Psychology and Methods, University of LeipzigLeipzig, Germany; ^4^Mind and Brain Institute, Charité and Humboldt UniversityBerlin, Germany; ^5^Faculty of Sport Science, Institute for General Kinesiology and Exercise Science, University of LeipzigLeipzig, Germany

**Keywords:** dynamic balance task, balance learning, healthy aging, non-invasive brain stimulation, kinematics, transcranial direct current stimulation

## Abstract

Older adults frequently experience a decrease in balance control that leads to increased numbers of falls, injuries and hospitalization. Therefore, evaluating older adults’ ability to maintain balance and examining new approaches to counteract age-related decline in balance control is of great importance for fall prevention and healthy aging. Non-invasive brain stimulation techniques such as transcranial direct current stimulation (tDCS) have been shown to beneficially influence motor behavior and motor learning. In the present study, we investigated the influence of tDCS applied over the leg area of the primary motor cortex (M1) on balance task learning of healthy elderly in a dynamic balance task (DBT). In total, 30 older adults were enrolled in a cross-sectional, randomized design including two consecutive DBT training sessions. Only during the first DBT session, either 20 min of anodal tDCS (a-tDCS) or sham tDCS (s-tDCS) were applied and learning improvement was compared between the two groups. Our data showed that both groups successfully learned to perform the DBT on both training sessions. Interestingly, between-group analyses revealed no difference between the a-tDCS and the s-tDCS group regarding their level of task learning. These results indicate that the concurrent application of tDCS over M1 leg area did not elicit DBT learning enhancement in our study cohort. However, a regression analysis revealed that DBT performance can be predicted by the kinematic profile of the movement, a finding that may provide new insights for individualized approaches of treating balance and gait disorders.

## Introduction

Due to the demographic change, the older population is constantly increasing. Therefore, understanding the mechanisms of aging processes and examining strategies to decelerate age-related decline is of great importance. One significant problem of the aging process is impaired motor ability (Smith et al., 1999; Krampe, 2002) which is the result of a complex interaction of peripheral and central processes involving losses in muscle strength/power (Frontera et al., 1991; McNeil et al., 2007) and alterations in the central nervous system (CNS; Burke and Barnes, 2006). Age-related decline in muscle strength most severely affects the dorsiflexor and extensor muscles of the lower extremities (Frontera et al., 1991; McNeil et al., 2007), which is why older adults often show increased postural sway (Baloh et al., 1994; Liaw et al., 2009). On the other hand, older adults, compared with younger adults, show a reduced amount of structural and functional brain plasticity (Burke and Barnes, 2006) and also learning-dependent plasticity decreases with age (Sawaki et al., 2003). All these factors contribute to an age-related decrease of postural stability, which is an important risk factor for falls (Granacher et al., 2008; Panel on Prevention of Falls in Older Persons, American Geriatrics Society and British Geriatrics Society, 2011). According to the Clinical Practice Guidelines for Prevention of Falls in Older Persons from 2011, exercise in the form of strength, balance, gait, and coordination training was shown to be effective in reducing falls in older adults (Panel on Prevention of Falls in Older Persons, American Geriatrics Society and British Geriatrics Society, 2011). Besides reducing the number of falls (Gardner et al., 2000), balance training also had a positive effect on gait and reduced the fear of falling (Wolf et al., 1996; Liu-Ambrose et al., 2004) and therefore is considered an effective intervention for treating age-related mobility losses. One study revealed that balance training is also capable of inducing neuroplastic changes in elderly but also in patients suffering from Parkinson’s disease (Sehm et al., 2014). Interestingly, these neuroplastic changes were correlated with the learning performance (Sehm et al., 2014) and therefore seem to be an important prerequisite for balance learning. Techniques of non-invasive brain stimulation have also been shown to induce neuroplastic changes and thereby also successfully facilitate task performance (Nitsche and Paulus, 2000; Madhavan and Shah, 2012). Transcranial direct current stimulation (tDCS) can facilitate motor performance by up-regulating neural activity in the underlying brain tissue (Nitsche and Paulus, 2000). Following on training studies attempting to enhance postural stability, recent studies have successfully targeted the leg area of the primary motor cortex (M1) by means of anodal tDCS (a-tDCS) to improve static balance (Dutta et al., 2014) and locomotion (Kaski et al., 2012) in young adults. A study in hemiplegic stroke patients showed that single-session tDCS can improve patients’ balance ability and increases the isometric strength of the affected lower extremity (Sohn et al., 2013). Therefore, combining the assessment of older adults balance ability with concurrent use of lower limb tDCS seems reasonable to evaluate the effect of brain stimulation on balance learning. In our previous study, the effect of tDCS on balance learning ability was investigated in healthy young subjects using a dynamic balance task (DBT; Kaminski et al., 2016). Our results showed that tDCS over M1 leg area is capable of enhancing balance performance in the DBT as participants showed higher task performance and lower error rates during and after tDCS compared to a control group. To follow up on these findings, in the present study we wanted to examine the feasibility of using the DBT as a balance learning task in elderly participants. Our main objective was to evaluate the effect of a-tDCS on DBT learning in older adults. Additionally, we analyzed the kinematic profile of DBT learning performance in our aged cohort as kinematic variables have been shown to be sensitive markers of postural stability (Yu et al., 2008; Bisson et al., 2014). In specific, we aimed to identify whether kinematic variables velocity, acceleration, jerk and postural sway frequency can predict balance performance in healthy elderly. In our previous study, we found that kinematic variables can well predict DBT performance in younger adults (Kaminski et al., 2013). Additionally, our results showed that performance improvements were mediated by tDCS-induced changes in movement velocity. According to this previous study, we hypothesized that (A) a-tDCS over M1 leg area during DBT learning facilitates learning performance compared with a group receiving s-tDCS in an older age cohort. Additionally, we expected that (B) the kinematic profile assessed during DBT learning predicts the DBT performance level of elderly with a special impact of velocity on performance improvement.

## Materials and Methods

### Participants

Thirty healthy elderly participants (17 females, mean age = 67.7 ± 6 years) were enrolled in this study. All participants gave written informed consent and the study procedures were approved by the local ethics committee of the University of Leipzig and conducted in accordance with the Declaration of Helsinki and only healthy participants were included. To exclude the presence of any neurological disease and/or contraindications, all participants underwent a detailed neurological examination prior to the testing phase. All participants were free of any medication affecting the CNS and were task naïve. All participants were right-handed as assessed by the Edinburgh Handedness Inventory (mean score 90.03; range 55–100; Oldfield, 1971) and did not show any signs of cognitive impairment, measured by the Mini Mental State Examination (MMSE, mean score: 29.23, range: 27–30; Folstein et al., 1975). Furthermore, we assessed participants standing balance ability before the experimental procedure using the Fullerton Advanced Balance (FAB) Scale (Rose et al., 2006), a multidimensional balance scale specifically designed to evaluate balance ability of functionally independent older adults. We also assessed participants’ level of physical activity with the long version of the International Physical Activity Questionnaire (IPAQ; Craig et al., 2003).

### Study Design

The study was comprised of two consecutive training sessions that were separated by 24 h. On the first training day (TD1), participants performed 15 trials of DBT training while 20 min of tDCS were applied over the leg motor cortex (M1 leg area). Participants were randomly assigned to either the experimental condition, where they received 20 min of a-tDCS, or the sham-control condition, where s-tDCS was applied. On the second training day (TD2), another 15 trials of DBT training were performed without tDCS. This was done to examine the effects of a-tDCS on consolidation of the newly acquired motor skill and to capture longer lasting a-tDCS effects on a consecutive training session. During each session, the platform position of each subject in each trial was continuously recorded using the Spike2 (Cambridge Electronic Design Ltd., Cambridge, UK) software.

### Whole-Body Dynamic Balancing Task (DBT)

The DBT was performed on a stability platform (model 16030, LaFayette Instruments, Lafayette, IN, USA) with a maximal deviation of 26° to each side. A detailed description of the procedure is provided elsewhere (Taubert et al., 2010; Kaminski et al., 2013). In brief, subjects were instructed to stand on the movable platform and to keep it in a horizontal position as long as possible during a trial length of 30 s. On each training day, 15 trials were performed with between-trial rest intervals of 90 s to avoid muscle fatigue. Hence, each training session lasted approximately 29 min, including breaks. To prevent falls, participants were secured with a safety harness during training. The primary performance measure was the total time participants were able to keep the platform in a horizontal position within a range of ±3° to each side, henceforth referred as Time in Balance (TiB). After each trial, participants were provided with their TiB value as verbal feedback but besides that, no strategy on how to best perform the task was provided (discovery learning approach; Wulf et al., 2003; Orrell et al., 2006).

### Transcranial Direct Current Stimulation (tDCS)

For tDCS, a weak direct current of 1 mA was delivered for 20 min using a battery driven stimulator (neuroConn GmbH, Ilmenau, Germany). On TD1, either a-tDCS or s-tDCS was applied to the bilateral M1 leg area during the first 10 trials of DBT performance. While the anode (5 cm × 5 cm) was placed over the M1 leg area target region, the cathode (reference electrode) was placed over the right frontal orbit (10 cm × 5 cm). The anatomical landmark for M1 leg area was chosen according to the 10–20 system and the anode was placed 1 cm behind the vertex on the mid-sagittal line (Madhavan and Stinear, 2010; Laczó et al., 2014). TDCS was applied using a highly conductive electrode paste (Ten20 CONDUCTIVE Neurodiagnostic Electrode Paste, Weaver and Company) and flexible elastic straps were used to fixate the electrodes on the head. Current was ramped up for 30 s in the beginning of tDCS eliciting a transient tingling sensation on the scalp that faded over seconds (Nitsche et al., 2003; Gandiga et al., 2006) and also ramped down for 30 s. During s-tDCS, the current was increased, maintained and decreased for 30 s each. Before and after tDCS, participants rated their level of attention (1 = not attentive, 10 = very attentive), fatigue (1 = very tired, 10 = not tired at all) and discomfort (1 = no discomfort, 10 = strong discomfort) on a visual analog scale (VAS).

### Data Analysis

For detailed data analysis, we recorded the platform position of each subject in each trial. This was done by transforming voltage to an amplifier that translated the signal into a Spike waveform at 5000 Hz. Before parameters were calculated, data was preprocessed using custom-built scripts in MATLAB version 8.2 (see also Kaminski et al., 2016). Data preprocessing included low-pass-filtering at 5 Hz using a 2nd order low-pass Butterworth filter to remove hardware derived artifacts and data resampling to 500 Hz. TiB was then calculated by calculating the total time per trial, subjects spent within a range of ±3° to each side. Additionally, we aimed to decode the kinematic profile of DBT performance using additional variables. Therefore, we calculated the first, second and third derivatives of position representing velocity, acceleration, jerk/smoothness. In addition, the number of zero crossings (ZC) was calculated as the total number of times that signal passed from one side of the horizontal position (0°) to the other. Statistical analyses were performed using IBM SPSS version 22.

#### Demographics

An independent-samples *t*-test was performed on each demographic variable (age, MMSE-score, total score of physical activity in IPAQ, total score in FAB-scale) to exclude that potential group differences in demographic variables might have influenced task performance. Repeated-measures analysis of variance (RM-ANOVA) with factor GROUP (a-tDCS, s-tDCS) and TIME (pre-post training) were used to assess changes in VAS scores.

#### Performance Measure Time in Balance (TiB)

Performance data was tested for normality using the Shapiro-Wilk test. As the test showed that the data was not normally distributed, non-parametric tests were used to examine learning improvement.

##### Training day 1

The Mann-Whitney U Test (MWU) was performed to assess baseline (trial 1) differences between groups. Overall learning was evaluated by performing a Friedman Test on factor TRIAL. First training session improvements were divided in online (trial 1–10) and offline (trial 11–15) learning improvements to disentangle acute tDCS effects and immediate tDCS after effects. Absolute improvement was calculated by subtracting last trial performance from first trial performance (online: t10–t1, offline: t15–t11) and compared between groups using the MWU. Additionally, percentage performance improvement was calculated by subtracting participants first trial performance (t1) from performance of training trial 15 and normalizing the difference to t1 performance and multiplying the term by 100 to create percentage values (online: (t10−t1)/t1*100, offline: (t15−t11)/t11*100). Percentage improvements were compared between groups using the MWU.

##### Consolidation and training day 2

To investigate skill consolidation from TD1 to TD2, the retention score, calculated as the difference between t15 TD1 performance and t1 TD2 performance, was compared between the two groups using MWU. Overall learning was evaluated by performing a Friedman Test on factor TRIAL. Absolute and percentage performance improvement on TD2 was calculated analogous to TD1 and compared between groups using the MWU.

#### Predicting DBT Performance by Kinematic Variables

##### Multiple regression

A partial correlation was performed between each kinematic variable and performance variable TiB. Subsequently, all variables having significant relations with TiB were entered in a regression model. The regression analysis was performed to decode the contribution of specific kinematic variables on overall DBT performance and thereby unravel the predictive power of the kinematic profile for balance performance measures. All variables were log-transformed before entering them into the model, thereby we created residuals with a normal distribution. In the regression model, TiB was defined as the dependent variable and the kinematic variables velocity, acceleration, ZC and trial were predefined as predictor variables. All main variables were entered at the same time and a full-model fit was examined.

##### tDCS effects on kinematic variables

To evaluate the effect of tDCS on our kinematic data, we calculated the absolute and percentage change of each variable analogous to our absolute and percentage improvement calculation (absolute: t15–t1, percentage: (t15–t1)/t1*100) and compared these values between groups using MWU.

For all analyses, a *p*-value of < 0.05 was considered to be significant.

## Results

### Demographics

There were no significant between-group differences in age (independent-samples *t*-test, *t*_(28)_ = −0.82, *p* = 0.42), MMSE-score (independent-samples *t*-test, *t*_(28)_ = 0.63, *p* = 0.53), total amount of physical activity (independent-samples *t-test*, *t*_(28)_ = 0.85, *p* = 0.4) or balance ability on the FAB-scale (independent-samples *t*-test, *t*_(28)_ = 0.13, *p* = 0.9); see also Table 1 for mean values of all variables). All participants tolerated the stimulation well. None of the participants reported any side effects from tDCS stimulation but most experienced the tingling sensation on the skin during the ramp-up phase of tDCS. Groups did not differ in their level of attention (RM-ANOVA Time × Group interaction, TD1: *F*_(1,28)_ = 2.87, *p* = 0.1, TD2: *F*_(1,28)_ = 1.26, *p* = 0.27), fatigue (RM-ANOVA Time × Group interaction, TD1: *F*_(1,28)_ = 0.19, *p* = 0.67 TD2: *F*_(1,28)_ = 0.05, *p* = 0.98) or discomfort (RM-ANOVA Time × Group interaction, TD1: *F*_(1,28)_ = 0, *p* = 1, TD2: *F*_(1,28)_ = 0.2, *p* = 0.89) before and after each of the DBT training days (see also Table 2 for mean values).

**Table 1:** **Group demographics**.

Group	Age (years)	MMSE	IPAQ	FAB
a-tDCS, *n* = 15	66.8 ± 5.63	29.33 ± 0.72	6855.6 ± 5682	36.67 ± 2.72
s-tDCS, *n* = 15	68.6 ± 6.00	29.13 ± 0.99	5383.8 ± 3590	36.53 ± 3.02

*MMSE: Mini Mental State Examination, total score range of 1–30; cut-off score for exclusion: ≤26. IPAQ: International Physical Activity Questionnaire, total score of physical activity in metabolic equivalent of task minutes per week (MET-minutes), cut-off for high level of physical activity per week: ≥3000. FAB: Fullerton Advanced Balance Scale, total score range of 1–40, cut-off score for higher risk of falls: ≤25. All values are depicted as mean ± standard deviation of the mean*.

**Table 2:** **Visual analog scale (VAS)**.

	TD1		TD2	
	Before	After	Before	After
**a-tDCS**
Attention	9.47 ± 0.74	9.60 ± 0.74	9.27 ± 1.16	9.40 ± 1.18
Fatigue	9.40 ± 1.12	9.47 ± 0.92	9.60 ± 0.74	9.67 ± 0.72
Discomfort	1.20 ± 0.56	1.20 ± 0.56	1.13 ± 0.35	1.20 ± 0.50
**s-tDCS**
Attention	9.53 ± 0.74	9.33 ± 1.18	9.47 ± 0.92	9.40 ± 1.12
Fatigue	9.27 ± 1.03	9.28 ± 0.98	9.27 ± 1.44	9.33 ± 1.40
Discomfort	1.13 ± 0.52	1.20 ± 0.53	1.27 ± 1.04	1.21 ± 0.99

*Attention, fatigue and discomfort were assessed on a VAS before and after the dynamic balance task (DBT) was performed on TD1 and TD2. Attention scale, ranging from 1 (no attention) to 10 (highest attention level). Fatigue scale, from 1 (high fatigue level) to 10 (no fatigue). Discomfort scale, ranging from 1 (no discomfort) to 10 (highest level of discomfort). All values are expressed as mean ± standard deviation. Please note that there were no changes in attention, fatigue or discomfort within groups (Before vs. After) or between groups (a-tDCS, s-tDCS) on TD1 or TD2*.

### Performance Measure Time in Balance (TiB)

#### Training Day 1 (TD1)

**Figure 1:**
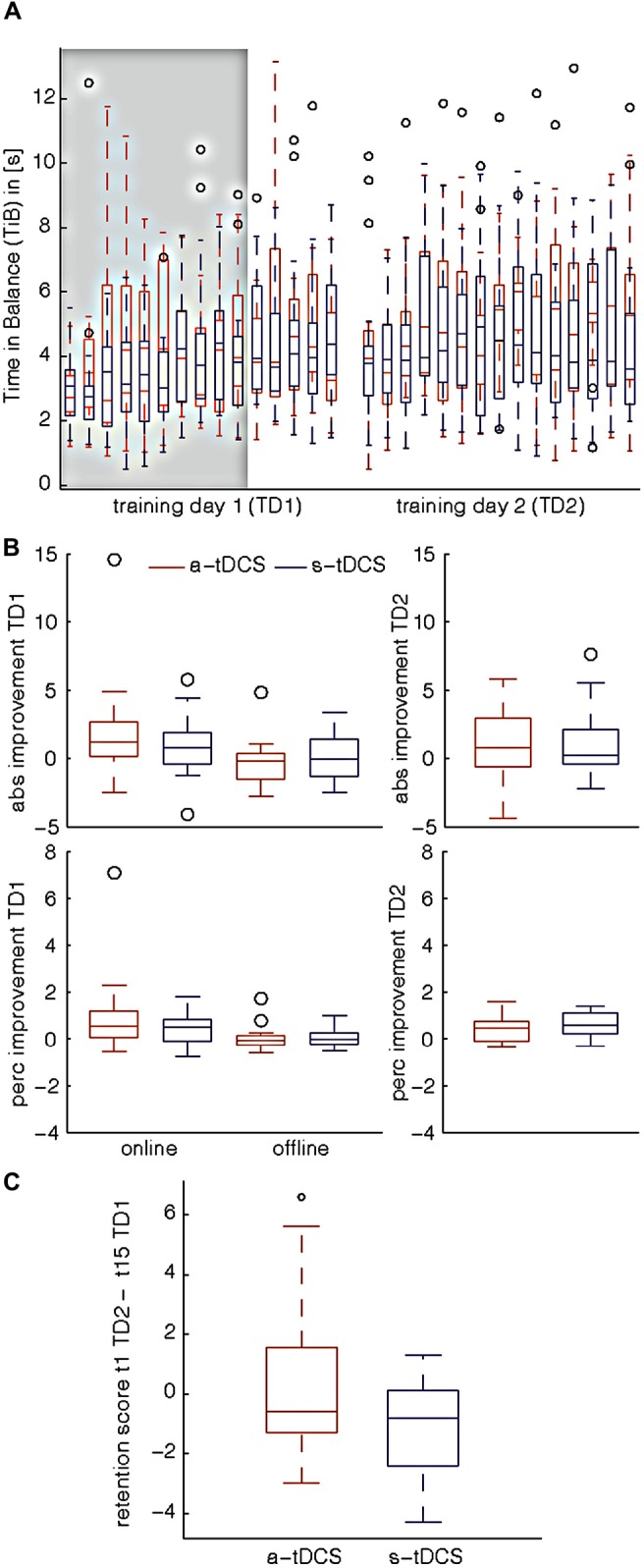
**Dynamic balance task (DBT) performance.** Results are shown for Training Day 1 (TD1) and Training Day 2 (TD2), which were separated by 24 h. a-tDCS: anodal tDCS, s-tDCS: sham tDCS, abs improvement: absolute improvement, abs improvement TD1: online improvement: trial10–trial1, offline improvement: trial15–trial11, TD2: trial15–trial1, perc improvement: percentage improvement, weighted difference of first and last trial performance multiplied by 100, perc improvement TD1: online improvement: ((t10−t1)/t1*100), offline improvement: ((t15−t11)/t11*100), TD2: ((t15−t1)/t1*100), retention score: difference between trial 15 TD1 and trial 1 TD2 performance (t15TD1–t1TD2). **(A)** Behavioral results for Time in Balance (TiB) performance on both training sessions. There was no baseline difference in TiB between the two groups (trial1, TD1) which indicates that all participants started at the same performance level. Both study groups significantly improved their level of performance over time on TD1 as well as on TD2. Gray shaded box indicates the time of a-tDCS/ s-tDCS stimulation. **(B)** Absolute/Percentage Improvement for TD1. No significant differences between a-tDCS and s-tDCS group were observed when comparing their absolute or percentage improvement gain. On TD1, neither online (t1–10) nor offline effects (t11–15) of tDCS showed a significant group difference. Therefore, one can conclude that the concurrent application of tDCS over M1 leg area did not elicit DBT performance enhancement in our study cohort **(C)** Retention score. There was no significant difference regarding the retention scores of the two groups, which indicates that tDCS did not affect skill retention from TD1 to TD2.

There was no baseline difference in TiB between the two groups (MWU: *U* = 101.5, *p* = 0.65), indicating that all participants started at the same performance level. Both groups significantly improved their DBT performance over time (Friedman: $\chi_{\left(14\right)}^{2}$ = 51.81, *p* < 0.001, Figure 1A). TiB under a-tDCS increased from 2.87 ± 1.09 s at baseline to 3.9 ± 1.74 s, while TiB under s-tDCS increased from 3.13 ± 1.22 s to 5.22 ± 2.77 s. We did not find significant differences between groups regarding their absolute performance improvement neither during tDCS stimulation (online effect, MWU: *U* = 106, *p* = 0.78) nor immediately after tDCS stimulation (offline effect, MWU: *U* = 85, *p* = 0.25). We also did not find significant group differences regarding percentage improvement gain neither during tDCS (MWU: *U* = 105, *p* = 0.76) nor after tDCS (MWU: *U* = 103, *p* = 0.69; see also Figure 1B).

#### Consolidation and Training Day 2 (TD2)

When comparing the retention scores of the two groups, we found no significant difference (MWU: *U* = 76, *p* = 0.13), which indicates that a-tDCS did not affect skill retention from TD1 to TD2 (Figure 1C). Similar to TD1, DBT-learning in both groups improved over time (Friedman: $\chi_{\left(14\right)}^{2}$ = 34.68, *p* = 0.002, Figure 1A). However, no significant difference regarding the absolute (MWU: *U* = 110, *p* = 0.94) or the percentage improvement gain (MWU: *U* = 109, *p* = 0.9) of the two groups was detected (Figure 1B). TiB increased from 4.24 ± 2.87 s to 5.33 ± 2.82 s under a-tDCS, while performance under s-tDCS increased from 3.5 ± 1.03 s to 5.05 ± 3.35 s.

### Relationship between Kinematics and Performance

#### Multiple Regression

**Figure 2:**
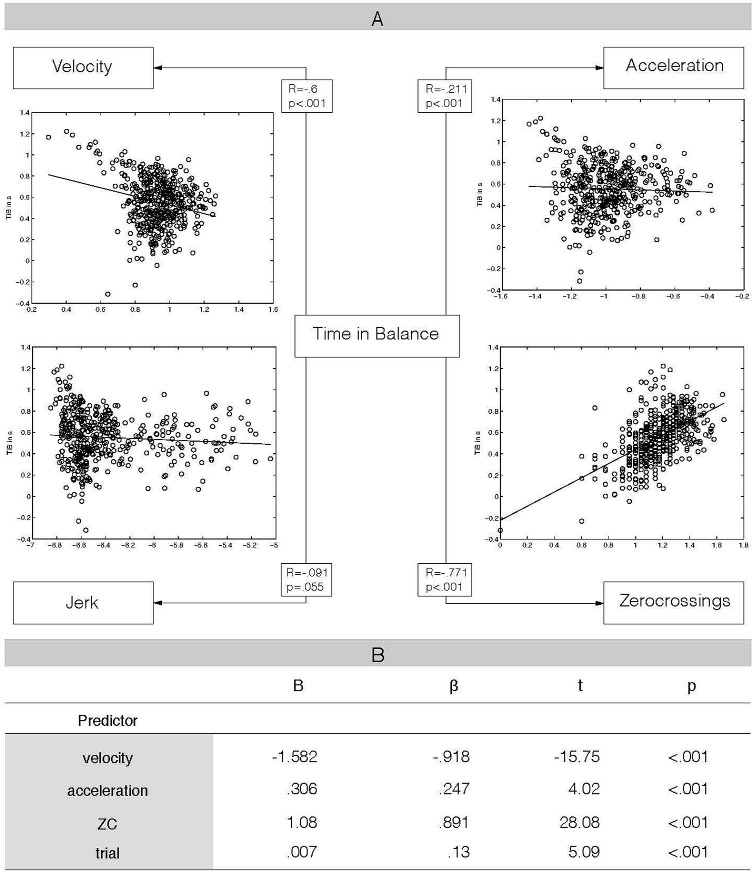
**Relationship between kinematic variables and balance performance**. **(A)** Results of partial correlation analysis, controlling for the other three kinematic variables, respectively. This analysis revealed a specific relationship between time in balance performance and kinematic variables velocity, acceleration and the number of zero crossings (ZC), but not for jerk. Additionally, scatterplots of the relationships between kinematics and performance are added. **(B)** Results from multiple regression analysis, Time in Balance (TiB) as dependent variable, velocity, acceleration, number of ZC and trial as independent predictors. ZC: number of zero crossings, B: unstandardized regression coefficient, β: standardized regression coefficient, *t* = *t* test value (*t*-statistic), *p* = *p*-value of *t*-statistic. Our multiple regression analysis revealed a significant relationship between each of the included variables and our dependent variable TiB. The kinematic variable velocity was negatively correlated with performance, while acceleration and ZC showed a positive relation with TiB. Trial was also positively correlated with TiB.

Figure 2A depicts the significant partial correlations between our dependent variable TiB and the kinematic variables velocity, acceleration and the number of ZC. As there was no significant correlation between TiB and jerk (see Figure 2A), we did not include jerk as a factor in the model. All other kinematic variables and variable trial were included as predictors in the model. The regression model revealed that each independent variable was significantly related to the dependent variable TiB (adjusted *R*^2^ = 0.72, *F*_(4,443)_ = 285.71, *p* < 0.001). Larger ZC- and larger acceleration values were associated with greater TiB values (positive correlation), while lower velocity values were associated with higher TiB values (negative correlation). Additionally, TiB and trial showed the expected positive association, indicating that TiB increased with ascending trial numbers (see also Figure 2B for regression weights).

#### tDCS Effects on Kinematic Variables

We did not detect any group difference regarding absolute or percentage change from t1 to t15 in our kinematic variables velocity (abs: *U* = 93, *p* = 0.42, perc.: *U* = 103, *p* = 0.69), acceleration (abs: *U* = 78, *p* = 0.15, perc.: *U* = 80, *p* = 0.18) or the number of ZC (abs: *U* = 100, *p* = 0.6, perc.: *U* = 105, *p* = 0.76). Interestingly, we found a significant group difference in jerk for absolute (*U* = 46, *p* = 0.006) as well as percentage change (*U* = 52, *p* = 0.012) from t1 to t15, even though the change in jerk was relatively small. While the a-tDCS group showed a small increase in jerk values from t1 to t15 (absolute change: 0.0002 m/s^3^, percentage change: 18%), jerk in the s-tDCS group rather decreased from t1 to t15 (absolute change: −0.0035 m/s^3^, percentage change: −28%). However, our partial correlation analysis did not reveal a significant association between jerk and TiB; therefore we assume that the group difference in jerk change did not affect our global learning measure TiB.

## Discussion

In the present study, our main objective was to examine whether a-tDCS over M1 leg area is capable of enhancing DBT learning in healthy elderly. We expected that, analogous to a younger cohort (Kaminski et al., 2016), targeting the M1 leg area region by means of tDCS would support DBT learning in the elderly and translate into superior learning performance compared to sham stimulation. However, even though both groups successfully increased their level of DBT performance over time, in the present study we found no difference between the a-tDCS and s-tDCS groups’ amount of learning. Thus, our results indicate that the application of tDCS over M1 leg area during DBT performance did not elicit performance enhancement in our aged study cohort. But the results also indicate that older adults are able to perform the DBT (Sehm et al., 2014) and significantly improve their performance even within a single training session. Future studies can use this knowledge to directly compare DBT performance between different age cohorts to further investigate age-related deficits in balance learning ability and identify related neural correlates using combined neurophysiological assessments of brain activation with behavioral outcome measures. Furthermore, our results show that a large amount of variance in DBT learning performance can be predicted by kinematic variables, a result that can particularly be important when diagnosing and treating balance and gait disorders.

### No Effects of tDCS on Complex Balance Learning in Elderly

Several studies have shown beneficial effects of tDCS over M1 leg area on postural control and locomotion. While Kaski et al. (2012) showed enhanced motor adaptation aftereffects in healthy young adults after using tDCS over M1 leg area, Sohn et al. (2013) were able to demonstrate that tDCS enhanced the overall stability index of hemiplegic stroke patients after only a single session (Sohn et al., 2013). In a previous study, we found that tDCS over M1 leg area does elicit performance improvements in DBT learning in a younger study cohort (Kaminski et al., 2016). However, using the same parameters of tDCS stimulation in our older study sample, we did not see behavioral improvements in DBT learning, a finding that might indicate age-related differences in the capacity for tDCS-induced behavioral changes. One potential explanation for this discrepancy is that the brain regions that are involved in initial DBT learning differ between younger and older adults. In a recent study, it was demonstrated that a single DBT training session results in cortical thickening of M1 leg area in healthy young adults (Taubert et al., 2016). This finding suggests a specific involvement of the leg area sub-region of M1 during initial DBT learning in a younger cohort. However, nothing is known so far about brain activation changes after a single DBT session in older adults. It might be that brain regions other than M1 leg area are more important during initial DBT learning in older age. Another issue that needs to be considered is the timing of the stimulation. It is known that neurophysiological effects of tDCS differ between older and younger adults with older adults showing delayed plasticity of M1 (Fujiyama et al., 2014). Therefore, future studies should consider applying tDCS in older adults before a motor task is performed. What is also known is that older adults exhibit more elaborate brain activation than younger controls when performing a motor task, potentially to compensate for an age-related decline of neuronal efficacy (Heuninckx et al., 2008). Therefore, one could argue that stimulating a single brain region by means of tDCS may not have been sufficient to activate the whole large-scale network responsible for successful performance of this complex task in older adults. This would also be in line with previous studies demonstrating that older individuals show different responses to non-invasive brain stimulation protocols as compared with younger adults (Müller-Dahlhaus et al., 2008; Ridding and Ziemann, 2010). It is also known, that the effects of tDCS on motor outcomes are highly variable (Horvath et al., 2016) and tDCS effects also vary across sessions and individuals (Chew et al., 2015). Additionally, large differences in brain structure as well as in brain function exist in older adults (Stewart et al., 2014), which may also affect task performance and responsiveness to tDCS protocols. Therefore, it may be that the inter-individual variability in older adults is greater than that of younger adults, and may impair our ability to detect differences between groups.

Taken together, we are not able to determine which factor or combination of factors, if any, may have contributed to a facilitatory effect of tDCS on DBT performance in older adults. As argued above, the outcome of tDCS is affected by multiple factors involving task characteristics and individual determinants (Ridding and Ziemann, 2010) and little is known about neuronal correlates of DBT performance in older adults. Therefore, more research is needed to draw a comprehensive picture on dynamic balance ability in the elderly and how non-invasive brain stimulation techniques may interact with such complex coordinative behavior.

### Relationship between Kinematic Variables and Balance Control

Previous studies suggest an association between changes in postural control and changes in parameters of movement kinematics. Our regression results are in line with previous findings, showing that the kinematic parameters velocity (Jeka et al., 2004), acceleration (Jeka et al., 2004; Yu et al., 2008) and information on postural sway (Manor et al., 2010) provide important information for maintaining postural control. While velocity showed a strong negative relationship with performance, acceleration and performance were weakly positively correlated. Both velocity and acceleration seem to be sensitive markers of postural stability (Yu et al., 2008), however, we also found that the number of ZC, reflecting postural sway speed, were strongly positively correlated with performance. It has already been shown that greater velocity is associated with higher center of pressure deviation and, thereby, lower postural control (Paillard, 2012). Postural sway velocity, especially in the medial–lateral direction, has high predictive value for individual fall risk (Bigelow and Berme, 2011). As slowing down walking speed can also be an effective strategy to reduce the risk of falls (Roos and Dingwell, 2013), our finding that lower velocity values are associated with higher DBT performance is in good agreement with the literature. Given that DBT learning is associated with higher-frequency movement adjustments, the positive relationship between the postural sway speed and performance is an indicator of greater movement automaticity (Wulf and Lewthwaite, 2009) and therefore also a marker for learning. Taken together, our data suggest that DBT learning performance can be predicted by the kinematic profile of the movement. This result could be of functional relevance for diagnostics of balance related disorders or individualizing gait retraining or fall prevention treatments.

In a second analysis, we aimed to evaluate the effect of tDCS on our kinematic data. Interestingly, we found a significant effect of tDCS on absolute and percentage change in jerk on training day 1, suggesting that a-tDCS resulted in a small increase of jerk values, while during s-tDCS, jerk was decreased. Higher jerk values reflect “jerkier” movements with more deviation in motion, while lower jerk values represent movements with higher smoothness. However, as we found no correlation between jerk and TiB, we assumed that the effect on jerk did not affect our global learning parameter TiB. While some studies suggested that a decrease in jerk is associated with better performance (e.g., James, 2014), another study rather stated a positive relation of higher jerk values and performance increase (Slaboda, unpublished data). As there are not many studies investigating jerk effects during highly complex, multi-joint movements, the effects of different jerk patterns remain ambiguous and have to be further explored.

### Balance and Aging

Older adults show reduced postural stability as declines in muscular strength most severely affect the lower extremities (Frontera et al., 1991; McNeil et al., 2007). However, postural instability also represents an insufficiency of attentional resources since maintaining posture requires the integration of many different modalities of information including vision, proprioception and vestibular feedback (Granacher et al., 2008). In daily life, posture is held during changing environmental conditions, thus making it necessary for the balance system to interact with an external dynamical system (Chagdes et al., 2013). It has been shown that interventions that focus on improving balance in the elderly are most effective when they incorporate more complex exercises (Halvarsson et al., 2015) and also involve cognitive components. The DBT provides a nice setting to evaluate balance ability in older adults as it forces the user to dynamically adjust posture to continuous changes in the environment, thus demanding high attentional resources as well as flexible adaptations. The unstable platform of the balance board nicely mimics continuous changes in the environment and thereby creates an ideal setting for evaluating and training complex postural behavior (Chagdes et al., 2013). Our results show that older adults are able to improve their balance ability during a single session of DBT training and maintain this motor skill at least until a second day of training. Therefore, using the DBT in a longer-term setting may support and improve classical fall prevention trainings and provide an interesting setup for training on instable platforms. However, even though we observed significant learning improvements, DBT learning curves of both groups were characterized by irregular increases and decreases of performance. The underlying mechanism remains unclear, however, one potential explanation might be the difficulty of the motor task. Since we wanted to maintain conditions from our younger study sample (Kaminski et al., 2016), older adults were tested with a TiB range of only 3° while in our previous study, 5° of TiB range were tested (Sehm et al., 2014), thus making it easier for older adults to meet the criterion for successful performance. Therefore, the task may have been more difficult and irregularities in performance may represent difficulties in maintaining better performance over the time course of the training session. On the other hand, decreases in TiB performance may be the result of muscle or cognitive fatigue since the DBT is both physically and attentionally demanding. This, however, seems rather unlikely, since changes in levels of attention but also both muscle and cognitive fatigue were assessed using a VAS and no significant changes were detected.

### Study Limitations

In the present study, we used behavioral measurements to assess motor learning in an aging population. However, since no neuroimaging measurements were included, it was not possible to investigate whether specific brain structures or specific brain states may have predicted DBT performance. Additionally, we cannot relate the variance in response to tDCS to a specific brain network. To get a better understanding of the neuronal correlates of DBT learning in older adults and potential tDCS effects on neuronal networks, further studies that combine neurophysiological assessments of brain activation with behavioral outcome measures are needed. Furthermore, our aged study cohort was selected according to relatively strict inclusion criteria and can therefore be considered healthy and active. In the long term, one goal would be to incorporate tDCS-usage as an add-on interventional strategy to treat balance and gait disorders; therefore older adults facing an increased risk of falls should be in the center of interest. Even though we did not detect any tDCS-induced effect on DBT performance in healthy older adults, it is possible that tDCS affects dynamic balance in patients. Additionally, we did not investigate the role of multiple tDCS-sessions on balance performance and did not test for any long-term effects. It is worth considering that multiple tDCS application sessions may have induced stronger behavioral effects that could be more persistent, as suggested by previous studies (Reis et al., 2009; Dell’Osso et al., 2011; Galletly et al., 2012). However, this study was the first step in understanding the role of single-session tDCS during the initial learning phase of a dynamic balancing task in older adults. While we provide initial evidence that tDCS over M1 leg area does not facilitate initial DBT learning in healthy older adults, future studies should be conducted investigating different time scales of DBT learning including also patient populations to draw a comprehensive picture of the effects of tDCS on dynamic balance performance.

## Conclusion

Combining measures of balance evaluation with methods of non-invasive brain stimulation in older adults is important to advance the knowledge on how to enhance treatment success in terms of fall prevention and gait training. Our results indicate that even though older adults are able to learn a dynamic balancing task over the time course of a single training session, concurrent application of tDCS over M1 leg area did not elicit DBT performance enhancement in our study cohort. More knowledge on neuronal processing of DBT learning in older adults, the influence of tDCS parameters, and the effect of inter-individual differences is required in order to draw a comprehensive picture of whether tDCS can help to enhance older adults dynamic balance learning. However, we also found that balance performance can be predicted by the kinematic movement profile, a result that could be of functional relevance to individualize gait retraining or fall prevention treatments for patients suffering from balance impairments.

## Author Contributions

EK: study design, study planning, execution, analysis, manuscript writing. MH: study execution, ideas for analysis, suggestions for manuscript writing. VR: study execution, suggestions for manuscript writing. CJS: data analysis, help with figure creation, suggestions for manuscript writing. CG: data analysis. BS: data analysis, suggestions for manuscript writing. AV: suggestions for manuscript writing, study design, interpretation of results. PR: corresponding author, study idea, hypotheses, study design, data analysis, manuscript writing.

## Conflict of Interest Statement

The authors declare that the research was conducted in the absence of any commercial or financial relationships that could be construed as a potential conflict of interest.

## Abbreviations

a-tDCS: anodal transcranial direct current stimulation
DBT: dynamic balance task
M1: primary motor cortex
s-tDCS: sham transcranial direct current stimulation
tDCS: transcranial direct current stimulation
TD: Training Day
TiB: Time in Balance
ZC: number of zero crossings.
